# On the Modulation of Brain Activation During Simulated Weight Bearing in Supine Gait-Like Stepping

**DOI:** 10.1007/s10548-015-0441-7

**Published:** 2015-07-24

**Authors:** Lukas Jaeger, Laura Marchal-Crespo, Peter Wolf, Andreas R. Luft, Robert Riener, Lars Michels, Spyros Kollias

**Affiliations:** Sensory-Motor Systems (SMS) Lab, Department of Health Sciences and Technology, ETH Zurich, ML G 59, Sonneggstrasse 3, 8092 Zurich, Switzerland; Clinic of Neuroradiology, University Hospital of Zurich, Zurich, Switzerland; Center of MR-Research, University Children’s Hospital, Zurich, Switzerland; Division of Vascular Neurology and Neurorehabilitation, Department of Neurology, University Hospital and University of Zurich, Zurich, Switzerland; Cereneo, Center for Neurology & Rehabilitation, Vitznau, Switzerland; Medical Faculty, Balgrist University Hospital, University of Zurich, Zurich, Switzerland

**Keywords:** Stepping, Foot loading, Body weight support, FMRI, Locomotion, MARCOS

## Abstract

To date, the neurophysiological correlates of muscle activation required for weight bearing during walking are poorly understood although, a supraspinal involvement has been discussed in the literature for many years. The present study investigates the effect of simulated ground reaction forces (0, 20, and 40 % of individual body weight) on brain activation in sixteen healthy participants. A magnetic resonance compatible robot was applied to render three different levels of load against the feet of the participants during active and passive gait-like stepping movements. Brain activation was analyzed by the means of voxel-wise whole brain analysis as well as by a region-of-interest analysis. A significant modulation of brain activation in sensorimotor areas by the load level could neither be demonstrated during active nor during passive stepping. These observations suggest that the regulation of muscle activation under different weight-bearing conditions during stepping occurs at the level of spinal circuitry or the brainstem rather than at the supraspinal level.

## Introduction

The role of the supraspinal sensorimotor areas in the control of muscle activation and sensory afferences associated with the bearing of body-weight during gait has not been understood in full detail. It has been shown that weight-bearing during upright standing as well as during the stance phase of walking activates a variety of load sensitive receptors located in the anti-gravity muscles of the legs (Dietz 1998; Duysens et al. 2000). Information about external forces acting upon the leg is fused in reflex pathways at the spinal cord level (Duysens et al. 2000). It has been suggested that the feedback from these load sensitive receptors is relayed to the central lumbosacral spinal circuitry. These central structures, i.e., the central pattern generators, provide the basic rhythmic patterns of muscle activation for the automated cyclic lower limb movements during upright human locomotion (Harkema et al. 1997).

While the timing of muscle activation determines interlimb coordination and hence the gait pattern, the degree of activation is critical for bearing of loads during walking. It has been shown that the amplitude of activity in anti-gravity leg muscles is inversely proportional to the amount of body-weight support (BWS) provided during treadmill walking (i.e., higher muscle activity for lower levels of BWS) (Ivanenko et al. 2002) (Dietz et al. 2002). It is plausible that anti-gravity muscles develop higher activity to account for increasing loads than ‘non-anti-gravity’ muscles when walking at lower levels of BWS. Patients with supraspinal lesions oftentimes present with hemiparesis impairing their gait (Bonita and Beaglehole 1988), and can only walk if BWS is provided. These observations may be explained by supraspinal control of the postural musculature.

Recent work using electroencephalography (EEG) during active treadmill walking has provided further evidence that supraspinal areas are indeed involved in the control of muscular activity during human gait. Several studies reported a modulation of activation in the primary sensorimotor areas (S1/M1) particularly during those phases of the stepping cycle which precede and succeed heel strike and toe off, i.e., the phases when loading and unloading of the lower limbs are imminent (Gwin et al. 2011; Wieser et al. 2010). The phase-dependent modulations in the supraspinal centers could thus be linked to preparing lower limb muscles for altering ground reaction forces during loading and unloading of the lower limbs. While these EEG studies during walking and stepping provided important insights into the temporal dynamics of the processes underlying the central drive of lower limb motor control, they did not specifically investigate the effect of walking under different levels of BWS. Since motor-related activity in the S1/M1 and the supplementary motor area (SMA) is highly correlated with muscular force output (Siemionow et al. 2000), it seems plausible that walking at different levels of BWS would also lead to a modulation of related neuronal activity. This assumption however, is challenged by two functional brain imaging studies investigating the supraspinal processes related to loading of the lower limbs during rhythmic multi-joint movements akin to human gait (Christensen et al. 2000; Miyai et al. 2006). Topographically, the reported activations are in rough agreement with the above-summarized EEG studies. However, during supine pedaling Christensen et al. did not find any correlation between the regional cerebral blood flow (CBF) in primary motor cortex and pedaling against different loads (0.5, 6, and 12 kg), using positron emission tomography (Christensen et al. 2000). In contrast, when compared to walking without any BWS, treadmill walking with BWS of 10 % of individual body weight (BW) led to a global signal increase in healthy participants and to a signal reduction in S1/M1 in patients with subcortical stroke as assessed by functional near-infrared spectroscopy (fNIRS) (Miyai et al. 2006).

In view of this inconclusive evidence and the methodological difficulties of the above mentioned studies (esp. the limited spatial resolution of fNIRS and EEG) further investigations on the physiology of motor control during weight bearing are justified.

The present study hence investigates whether a potential load related effect on brain activation is attributable to the integration of load related afferences, or rather to the generation of corresponding motor output. We use task-related fMRI combined with a MR-compatible stepper MARCOS, rendering different levels of external loads to the sole of the feet during supine gait-like stepping movements (Hollnagel et al. 2011; Jaeger et al. 2014). Passive (i.e., performed by the stepping robot) as well as active movements (i.e., performed by the participant) are investigated. If a modulation of brain activation is associated with the amount of generated lower limb muscle force, different loads will result in significantly different levels of brain activation during active movements. If a modulation of brain activation occurs in response to modulated loads during passive movements, this would be an indication that brain activation is primarily driven by load related afferent feedback. We hypothesized that the fMRI blood-oxygen-level dependent (BOLD) signals in sensorimotor areas are significantly influenced by the level of load acting on the lower limbs during active but not during passive stepping.

## Methods

The study was approved by the Ethics Committee of the Canton of Zurich (approval Nr. 856) and was conducted in accordance with the Declaration of Helsinki. Participants were not included in the study if they met any of the following exclusion criteria: (1) diagnosed neurological, musculoskeletal or cardiac dysfunction at present or in the past, (2) cardiac pacemaker, neuro-stimulator, or hearing aid, and (3) drug-abuse. All participants were informed about the aims and the course of the study and gave written consent for their participation.

### MARCOS

The MR-compatible stepper MARCOS is a one-degree-of-freedom robotic device actuated by two pneumatic cylinders per leg (www.sms.hest.ethz.ch/research/mr_robotics/setup). All parts are made from materials of low magnetic susceptibility (i.e., aluminum, brass, polyvinyl chloride). The arrangement of the pneumatic actuators allows each leg to independently perform predefined flexion and extension movements in the sagittal plane. The resulting movement resembles ‘marching-on-the-spot’. The cylinder attached to each foot allows imposing an external load of up to 400 N per leg along the cranio-caudal body axis, that simulates ground reaction forces. The desired load at the foot is inversely proportional to the position of the knee, such that highest force levels occur at full extension of the leg. Therefore, when participants move the legs in a step-like manner, the resulting load profile is of sinusoidal shape. A sinusoidal force profile was chosen over the typical ‘double hunch’ during slow ground-level gait in order to limit excessive head motion during image acquisition. Movement kinematics and kinetics were measured and stored by built-in position and force sensors of the robot at a sampling frequency of 80 Hz for off-line analysis of participant and robot performance. A custom made hip-fixation, a vacuum pillow at the back of the participants, shoulder belts, and an inflatable pillow (Crania, www.pearltec.ch) around the head secured the torso and the head of the participants preventing excessive head motion. For a more detailed technical description of the robot please refer to (Hollnagel et al. 2011).

### Motor Paradigm

Data from 16 healthy participants were collected during *active* and *passive* stepping inside the MR-compatible stepper. *Active* and *passive* stepping conditions were measured at loads of 0 % (*load level 0*), 20 % (*load level 20*) and 40 % (*load level 40*) of individual body weight. The *stepping frequency* and *knee amplitude* were maintained constant across all load levels and conditions. FMRI data during stepping at each load level were acquired in a block design in six separate runs that were presented in random order [i.e., 2 conditions (*active/passive*) × 3 load levels (0/20/40)]. Each run consisted of 15 blocks of movement, and 15 blocks of a baseline control condition. Block duration was 10 s, interleaved by 9.075 s of image acquisition.

Movement frequency was paced to 0.5 Hz by the presentation of a metronome through ear phones as applied by others (Ciccarelli et al. 2005; Mehta et al. 2009). The metronome was also presented during passive movements, as well as during the control condition, in order to equal auditory input. The beginning of each trial was indicated on the screen located near the feet of the participants, either by the presentation of the word ‘MOVE’ for movement trials or ‘LISTEN’ for control trials. During the passive movement condition, participants should relax their legs and not engage in active leg flexion- and extension while the robot enforced a desired trajectory with predefined foot load profile, amplitude, and frequency. During the active condition, participants should voluntarily produce leg flexion and extension while the robot followed the movement of the participant and rendered the desired load against its feet. In this condition, the cylinders attached to the knees limited the amplitude of the movement, but not the frequency. In the control condition, participants were instructed to listen to the metronome, however neither any stepping movements nor any loads occurred in this part of the experiment. During image acquisition between ‘MOVE’ and ‘LISTEN’ trials, participants were instructed to fixate on a white cross presented at the center of the screen, and not to think about moving their legs when listening to the metronome in order to minimize effects of movement imagination or rehearsal. Participants were familiarized with active and passive stepping at the three load levels inside the robot prior to image acquisition. Before the start of each functional run, participants were informed about the type of condition (*active* or *passive*), and whether a load was going to be rendered. They were however not explicitly informed about the amount of the load.

### Image Acquisition

Imaging data of all participants were collected on a 1.5 T Philips Achieva scanner (Philips Medical Systems, Best, the Netherlands) at the University Hospital of Zurich using an 8-channel SENSE™ head coil. The sparse sampling imaging protocol consisted of clusters of image acquisition interleaved by silent gaps of 10 s length (Jaeger et al. 2014). Each imaging cluster comprised of three consecutive volumes (TR = 3.025 s). The duration between the onsets of two imaging clusters was hence 19.075 s. 93 volumes in 31 clusters of 3 volumes were acquired, using a whole brain T2*-weighted, single-shot, echo planar imaging (EPI) sequence (TE = 50 ms, flip angle = 90 °, SENSE factor = 1.6). 35 interleaved, angulated, transversal slices covering the whole brain were acquired in each volume (field of view = 220 mm × 220 mm, acquisition voxel size: 2.75 mm × 2.8 mm × 3.8 mm, re-sliced to 1.72 mm × 1.72 mm × 3.8 mm).

### Data Processing and Statistical Analysis

#### Motor Performance

Custom Matlab routines (Matlab 2012b, Mathworks Inc., Natick, MA, USA, www.mathworks.com) were used for offline analysis of task performance. Position sensor data were filtered (low pass 1st order Butterworth filter, cut-off frequency was set to 4 Hz), and subsequently position and load profiles were extracted in order to calculate the performance metrics *foot load*, *knee amplitude,* and *movement frequency* for each individual step of each leg and load level. *Foot load* was defined as the maximal interaction force between the foot and the robot during each single step. *Knee amplitude* was defined as the vertical range of motion of the knee, and *movement frequency* was defined as the number of steps of one leg per second. Within each participant and load level, values were then averaged across all steps and over both legs, as *foot load*, *knee amplitude*, and *movement frequency* values of the left and the right leg were not significantly different (paired-sample *t*-tests, all *p*-values > 0.1). Subsequently, participant means were entered into a one-way ANOVA with repeated measures to test for a significant main effect of load in each performance metric in both stepping conditions individually (α = 0.05). Post-hoc paired samples *t*-tests were calculated to reveal differences between load levels, a Bonferroni-correction was applied to correct for multiple comparisons.

Position and load profiles were resampled to a step cycle of 0–100 % and then averaged across the left and the right leg and over all steps of each individual participant per load level and condition.

#### FMRI Data

BOLD-imaging data analysis was conducted using SPM8 (Wellcome Department of Cognitive Neurology, London, UK, www.fil.ion.ucl.ac.uk/spm) running on Matlab 2012b (Mathworks, Inc., Natick, MA, USA, www.mathworks.com). For each run, the three volumes prior to the first ‘MOVE’ block were removed from the data. The remaining 90 images were realigned to the mean image and unwarped to account for residual head motion related variance and image distortions along air-tissue boundaries (Andersson et al. 2001). Images were normalized to standard MNI space using the EPI template provided by the Montreal Neurological Institute (MNI brain), re-sliced to 2 × 2 × 2 mm^3^ voxel size, and smoothed (FWHM = 8 mm). The estimated realignment parameter data were filtered using the discrete cosine transform matrix filter (cut off at 128 s) incorporated in SPM8, to remove linear baseline drifts. Only data of participants whose estimated head motion was below the stringent threshold of ½ voxel size after filtering in every direction in all three load levels and both conditions were taken to 1st level statistical analysis. For each condition, data of the three load levels were modeled as three separate regressors in one general linear model (GLM) (Friston et al. 1994) for each participant individually. The auditory control condition was not modeled explicitly. Two additional regressors of no interest were added to the GLM accounting for the T1-decay along the three consecutive volumes (Zaehle et al. 2007). A high pass filter (cut off at 128 s) was used to remove slow signal drifts. To account for the sparse-sampling fMRI scheme, data during each trial were modeled using a boxcar function [1st order, window length 3 × TR (i.e., 9.075 s)] (Liem et al. 2012). Contrast images were computed for *load level 0*, *load level 20,* and *load level 40* (all against an implicit baseline). The contrast images from the 1st-level analyses were then subject to the following statistical voxel-wise whole brain tests at the 2nd-level:

1. One sample *t*-test for each load level in each condition to test for differences between task execution and the (not explicitly modeled) auditory control condition.
2. Paired samples *t*-tests to investigate differences between *active* and *passive* stepping at *load level 0*. These tests were conducted to verify the results from our previous study (Jaeger et al. 2014).
3. One-way repeated measures ANOVA (rmANOVA) with the factor *load* in each condition to reveal a possible modulation of brain activation by the variation of load across levels.
4. Two-way rmANOVA with the factors *load* and *condition* to investigate potential interaction effects between factors, as well as possible main effects of *load* and *condition*.

All of the resulting maps were thresholded at a cluster-corrected voxel threshold of p < 0.001 (spatial extent: k ≥ 42 contiguous voxels) (Forman et al. 1995; Slotnick et al. 2003). The cluster threshold method was applied to control for the overall type I error. Anatomical correlates of activated clusters were determined using probabilistic cytoarchitectonic maps implemented in the Anatomy toolbox (Eickhoff et al. 2005).

Voxel-wise statistical testing was followed up by the region of interest (ROI) analysis to confirm the results from the whole brain analyses. Sensorimotor ROIs were defined according to our previous fMRI study, in which we compared brain activation during active and passive stepping without load variation (Jaeger et al. 2014): left secondary somatosensory cortex (S2) (-50/-32/20), right S2 (46/-30/24), and cerebellar vermis (0/-46/-8). Bilateral spherical ROIs (radius of 4 mm) using the spatial coordinates for knee movements from (Kapreli et al. 2006) were defined for left S1/M1 (-14/-37/65) and right S1/M1 (16/-35/67), SMAproper left at (-2/-24/66) and right at (0/-24/68), CMA left at (-12/-6/44) and right at (10/-6/42). Values of  % fMRI signal change were then extracted from each ROI and load level in each condition for all participants using the SPM toolbox ‘MarsBaR’ (Brett et al. 2002). Similar to the analysis at the whole brain level, we performed a paired *t*-test on the ROI-data from both conditions at *load level 0*, as well as the one-way and two-way rmANOVAs using the data from both conditions at all load levels.

The rmANOVAs at the whole brain level and at the ROI level were carried out to test our hypothesis that brain activation is modulated by load during active but not during passive movements. The hypothesis would be confirmed, if a significant effect of *load* was found in the one-way rmANOVA for the active condition and if a significant interaction effect of *load* × *condition* but not a significant main effect *load* was found in the two-way rmANOVA. All rmANOVAs were followed-up by post-hoc *t*-tests to reveal within factor differences.

## Results

Eight participants were excluded from further analysis due to excessive head motion (i.e., translation of more than ½ voxel size in any direction) in at least one of the load levels. The remaining eight participants (3 male) aged 24.75 (3.46) [mean (standard deviation)] years, weighed 69.94 (8.91) kg, and were  all right handed and footed (Elias et al. 1998) (Table 1). The participants of the present study are a subset of those reported in (Jaeger et al. 2014).

**Table 1:** Individual anthropometric data of the study sample

Participant	Age (years)	Sex	BW (N)	Absolute foot load (N) at load level			Body height (m)	WHQ	WFQ
				0	20	40			
1	22	F	569	0	113.8	227.6	169	14	3
2	24	F	725.9	0	145.2	290.4	170	16	4
3	24	M	784.8	0	157	313.9	181	16	17
4	23	M	750.5	0	150.1	300.2	180	16	16
5	22	F	539.6	0	107.9	215.8	166	16	19
6	33	M	745.6	0	149.1	298.2	170	16	11
7	23	F	745.6	0	149.1	298.2	170	15	10
8	27	F	627.8	0	125.6	251.1	165	16	8
mean (SD)	24.75 (3.46)	–	686.1 (87.4)	–	137.2 (17.5)	274.5 (34.9)	171.38 (5.57)	15.63 (0.7)	11 (5.57)

Group mean values and standard deviation (SD) can be found at the bottom of the table. Absolute foot loads (N) are the desired maximum loads to which the robot was pre-set at the beginning of each experiment. BW = body weight, WHQ = Waterloo Handedness Questionnaire, values may range from −16 to 16, WFQ = Waterloo Footedness Questionnaire, values may range from −20 to 20, positive values represent dominance of the right side of the body in both tests

### Motor Performance

Between 70 and 75 steps were entered into the analysis of participant motor performance in each individual load level and condition. The descriptive statistics of the performance parameters *knee amplitude, stepping frequency,* and *foot load* at the three load levels for both movement conditions *active* and *passive* can be found in Table 2.

**Table 2:** Descriptive statistics of measures of motor performance *foot* *load, stepping frequency*, and *knee* *amplitude* during active and passive stepping at the three different levels of foot loading

		Passive				Active			
	Load level	Mean	SD	Min	Max	Mean	SD	Min	Max
Foot load (%-BW)	0	6.69	1.44	5.36	9.00	9.34	3.06	6.47	16.16
	20	20.99	0.66	20.14	22.26	21.66	4.85	16.28	30.91
	40	35.48	4.29	31.40	41.97	34.11	2.73	30.70	37.39
Stepping frequency (Hz)	0	0.51	0.01	0.50	0.54	0.55	0.04	0.49	0.61
	20	0.51	0.00	0.50	0.51	0.54	0.03	0.51	0.59
	40	0.52	0.02	0.51	0.56	0.54	0.02	0.50	0.57
Knee amplitude (m)	0	0.14	0.00	0.14	0.15	0.16	0.03	0.10	0.19
	20	0.15	0.01	0.14	0.16	0.16	0.02	0.14	0.20
	40	0.15	0.01	0.14	0.16	0.15	0.02	0.13	0.18

Values for *foot load* are the maximal force values as measured by the force sensors at the foot fixation of the robot. n = 8, SD = standard deviation, min = minimum, max = maximum,  %-BW = percent body weight

**Fig. 1:**
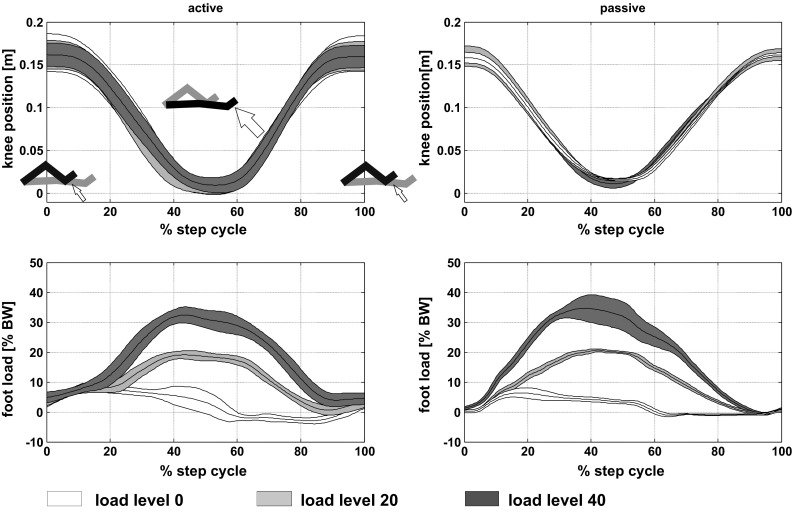
The *top row* shows group mean knee position profiles during active (*left*) and passive (*right*) stepping at the three load levels 0, 20, and 40. In the *top left* plot the black leg of the stick figure schematically represents the corresponding posture of the leg, the step cycle begins and ends with knee flexion. The* bottom row* shows the associated group averaged foot load profiles during active (*left*) and passive (*right*) stepping. The forces were measured in perpendicular to the sole of the foot, as indicated by the *white arrows* in the *top left*. The *center line* indicates the mean course, the *shaded area* represents mean ± one standard deviation, n = 8,  %-BW = percent body weight

The one-way rmANOVA calculated for the performance metrics *knee amplitude* and *stepping frequency* did not reveal a significant effect of *load level* in any of the stepping conditions *active* or *passive* (*knee amplitude* during *active*: F_2,14_ = 1.589, p = 0.239, and passive: F_2,14_ = 0.157, p = 0.856; *stepping frequency* during *active*: F_2,14_ = 0.271, p = 0.766, and *passive*: F_1.039,7.273_ = 1.983, p = 0.201 with a Greenhouse-Geisser correction). The group averaged *knee position* profiles largely overlap across the three load levels in both conditions, with higher variability during *active* than *passive* stepping (Fig. 1, top row).

For the performance parameter *foot load*, the one-way rmANOVA revealed a significant effect of *load level* in *active* (F_2,14_ = 92.155, p < 0.001) as well as *passive* (F_1.123,7.862_ = 384.666, p < 0.001, with a Greenhouse-Geisser correction) stepping. In both conditions post-hoc paired samples *t*-test revealed significant differences between all *load levels (active: load level 0* vs. *load level 20:* t_7_ = −5.734, p = 0.001; *load level 20* vs. *load level 40:* t_7_ = −7.495, p < 0.001; *load level 0* vs. *load level 40:* t_7_ = −15.331, p < 0.001; *passive: load level 0* vs. *load level 20:* t_7_ = −34.773, p < 0.001; *load level 20* vs. *load level 40:* t_7_ = −10.868, p < 0.001; *load level 0* vs. *load level 40:* t_7_ = −25.400, p < 0.001). The group averaged profiles of *foot load* across a step cycle show higher variability in two out of three load levels (0 and 20) for *active* than for *passive* stepping (Fig. 1, bottom row).

In both conditions, the desired loads deviated from the predefined values to a variable extent. *At load level 0,* these deviations amounted to 9.34 (3.06) %-BW during *active*, and to 6.69 (1.44) %-BW during *passive*, respectively. The desired values were reached with the highest accuracy at *load level 20* in both conditions. The measured values deviated on average only about 0.99–1.66 % from the targeted loads (*active*: 21.66 (4.85) %-BW; *passive:* 20.99 (0.66) %-BW). At load *level 40* the measured values were on average between 4.52 and 5.89 %-BW below the targeted values in both conditions (*active*: 34.11 (2.73) %-BW; *passive:* 35.48 (4.29) %-BW during). Despite these deviations from the predefined values, an average level-wise increase of approximately 12 %-BW (mean absolute value: 85.5 N) from one load level to the next in *active* stepping and 15 %-BW (mean absolute value: 98.5 N) in *passive* stepping was measured. From level 0 to level 40 a total mean increase of 171 N was observed during *active*, and 197 N during *passive* stepping, respectively.

### Brain Activation During Active and Passive Stepping at Different Load Levels

#### Voxel-Wise Whole Brain Analysis

**Fig. 2:**
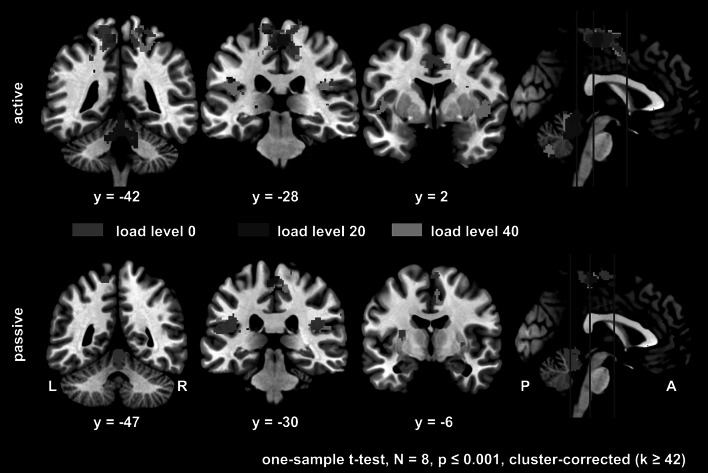
Overlay of areas of significant BOLD-signal increase during *active* (top) and *passive* (bottom) stepping at the *load levels 0* (*red*), *20* (*blue*), and *40* (*green*) as revealed by the 2nd-level group analyses (separate one-sample *t*-tests for each load level). The level of the coronal slices is indicated by the *blue lines* in the sagittal slice on the right. L = left hemisphere, R = right hemisphere, P = posterior, A = anterior, n = 8, p ≤ 0.001, cluster-corrected, k ≥ 42 consecutive voxels (Color figure online)

The one-sample whole brain *t*-tests at the 2nd-level revealed overlapping clusters of significant BOLD-signal increase during *active* stepping in bilateral medial S1/M1 and SMAproper at all three load levels. At *load level 0* this set of activations revealed additional bilateral activation of the cingulate motor area. At *load level 20*, the cerebellar vermis and the left thalamus were additionally activated. The most widespread set of regions was observed during *load level 40* including bilateral S2, the dorsal-posterior part of the anterior insula, left thalamus as well as the right superior and middle occipital gyri (Fig. 2, top row, and Table 3).

**Table 3:** Cortical and subcortical areas of significant peak BOLD-signal increase during the two conditions *active* and *passive* stepping at the three different levels of foot load 0, 20, and 40, as revealed by separate one-sample *t*-tests

Condition	Load level	Anatomy	Left hemisphere						Right hemisphere					
			Area	t	k_E_	x	y	z	Area	t	k_E_	x	y	z
Active	0	SMA-proper	–	–	–	–	–	–	4a	13.37	2283	12	−28	52
		Superior occipital gyrus	–	–	–	–	–	–	18	8.69	83	20	−92	24
	20	Vermis	–	–	–	–	–	–	–	36.26	625	8	−42	−24
		S1/M1	6	14.17	860	−10	−28	72	–	–	–	–	–	–
		Thalamus	–	9.1	72	−24	−18	14	–	–	–	–	–	–
	40	Anterior insula	–	7.21	45	−46	2	2	–	17.7	252	48	0	−2
		Vermis	–	8.45	63	−2	−68	−36	–	16.2	490	4	−48	−12
		Middle occipital gyrus	–	–	–	–	–	–	–	11.92	229	44	−72	6
		Precuneus	–	10.7	1498	−14	−38	58	–	–	–	–	–	–
		S2	OP1	9.55	122	−48	−28	22	OP2	8.92	58	36	−24	20
		Thalamus	–	8.58	71	−18	−24	6	–	–	–	–	–	–
		Superior occipital gyrus	–	–	–	–	–	–	–	7.14	52	18	−90	20
Passive	0	S2	IPC	21.59	383	−56	-26	18	IPC	11.19	172	38	−30	22
		Vermis	–	–	–	–	–	–	–	13.83	255	4	−48	−8
		Putamen	–	10.49	60	−28	−4	10	–	9.29	88	32	−4	2
		Precuneus	4a	9.62	380	−6	−40	70	–	–	–	–	–	–
		SMA-proper	–	–	–	–	–	–	6	8.41	307	4	−12	72
	20	S1/M1	–	–	–	–	–	–	4a	7.23	135	12	−26	58
	40	S2	IPC	14.57	116	−44	−32	22	OP1	10.91	138	46	−30	16
		SMA-proper	–	–	–	–	–	–	–	8.78	174	14	−26	54
		S1/M1	4a	6.59	77	−4	−28	54	–	–	–	–	–	–
		Precuneus	4a	5.86	46	−4	−40	66	–	–	–	–	–	–

S1/M1 = primary sensorimotor cortex, S2 = secondary somatosensory cortex, SMA = supplementary motor area, t = maximum* t* statistic, k_E_ = cluster size, voxel threshold is p ≤ 0.001, cluster corrected, k ≥ 42 consecutive voxels

*Passive* stepping elicited significant BOLD-signal increases in bilateral medial S1/M1 in all load levels, however, in contrast to *active* stepping the spatial extent of activated clusters did not overlap across loads. *Load level 0* additionally led to activation in bilateral SMA-proper, and S2 in the fronto-parietal operculum. Subcortical activations in bilateral putamen and vermis were also observed at this load level. Bilateral S2 and SMA-proper as well as right-sided CMA activations were also present at *load level 40* (Fig. 2, bottom row and Table 3).

The paired samples *t*-test between *active* and *passive* stepping at *load level 0* did not reveal any significant differences between the two conditions when applying a threshold of p ≤ 0.001 (cluster corrected at k = 42 consecutive voxels). However, at p ≤ 0.005 (cluster corrected at k = 70 consecutive voxels), significantly higher activation in the anterior and posterior cingulate cortex bilaterally as well as in the left lateral parietal cortex and putamen were found during *passive* than during *active* stepping. In the opposite contrast of *active* versus *passive* significantly higher bilateral activation in the cerebellum was observed at the same threshold.

In the whole brain voxel-wise one-way rmANOVA for the condition *passive*, a significant main effect of *load* was found in a cluster covering the left angular gyrus (F_2,14_ = 23.43, p < 0.001). Post-hoc paired samples *t*-test between all load levels revealed significantly higher activation in the angular gyrus bilaterally at *load level 0* than at *load level 40*. No significant differences were found in any of the other post-hoc comparisons in this condition.

For the condition *active*, a significant main effect of *load* was found in the one-way rmANOVA in a cluster covering the right middle occipital gyrus (F_2,14_ = 23.46, p < 0.001). Post-hoc hoc paired samples *t*-tests between all load levels revealed significantly higher activation during *load level 0* than during *load level 20* in the right angular gyrus and superior frontal gyrus.

The whole brain voxel-wise two-way rmANOVA did not reveal a significant interaction effect of *load* by *condition*. A significant main effect of *condition* was found in an extensive cluster located in the cerebellum (vermis and both hemispheres) (F_1,35_ = 33.00, p < 0.001) with higher average activation during *active* than during *passive* movements in this area. A significant main effect of *load* was found in the right hippocampus (F_1,35_ = 27.53, p < 0.001).

#### ROI-Analysis

The paired *t*-tests between the mean  %-signal change during *active* and *passive* stepping at *load level 0* revealed a trend of significantly higher activation in S1/M1 during *active* than during *passive* stepping (t = 2.036, p = 0.081). No significant differences or trends were found in any of the other investigated ROIs.

The one-way rmANOVA during *passive* stepping did not reveal a main effect of *load* in any of the investigated ROIs (Vermis: F_2,14_ = 0.348, p = 0.712. left S2: F_2,14_ = 1.008, p = 0.390, right S2: F_2,14_ = 0.612, p = 0.556, S1/M1: F_2,14_ = 0.063, p = 0.939, CMA: F_2,14_ = 1.754, p = 0.209, SMAproper: F_2,14_ = 0.446, p = 0.649).

The one-way rmANOVA during *active* stepping did not reveal a main effect of *load* in any of the investigated ROIs (Vermis: F_2,14_ = 1.705, p = 0.217, left S2: F_2,14_ = 0.553, p = 0.588, right S2: F_2,14_ = 0.966, p = 0.404, S1/M1: F_2,14_ = 2.539, p = 0.115, CMA: F_2,14_ = 0.899, p = 0.429, SMAproper: F_2,14_ = 0.123, p = 0.885).

**Fig. 3:**
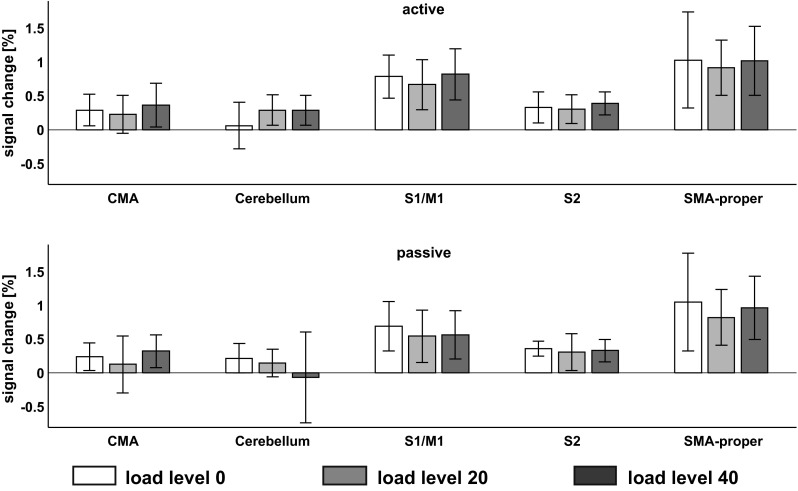
Percent signal change during *active* (top row) and *passive* (bottom row) stepping across the *load levels 0*, *20*, and *40* extracted from the regions of interest (ROI) as labeled on the abscissa. No effect of "load" was found in any of the examined ROIs. Spherical ROIs with a radius of 4 mm were created from peak coordinates for knee and ankle movements reported by (Kapreli et al. 2006). *Bar height* indicates the groups mean,* error bars* are ± one standard deviation. CMA = cingulate motor area, S1/M1 = primary sensorimotor cortex, S2 = secondary somatosensory cortex, SMA-proper = supplementary motor cortex proper, n = 8

The two-way rmANOVA did not reveal a significant *load* by *condition* interaction in any of the ROIs (Vermis: F_2,14_ = 0.041, p = 0.960, left S2: F_2,14_ = 0.179, p = 0.838, right S2: F_2,14_ = 0.041, p = 0.960, S1/M1: F_2,14_ = 0.851, p = 0.448, CMA: F_2,14_ = 0.383, p = 0.688, SMAproper: F_2,14_ = 0.313, p = 0.736). A significant main effect of *condition* was found in the vermis, with higher mean values during *active* than *passive* stepping (F_1,7_ = 12.666, p = 0.009), and a trend for a significant effect of *condition* was found in S1/M1 (F_1,7_ = 3.363, p = 0.1), again with higher average activation during *active* than *passive* movements. In the other ROIs, no significant effect of *condition* was found (left S2: F_1,7_ = 0.036, p = 0.855, right S2: F_1,7_ = 0.496, p = 0.504, CMA: F_1,7_ = 0.774, p = 0.408, SMAproper: F_1,7_ = 0.688, p = 0.434). No main effect of *load* was found in any of the ROIs (Vermis: F_2,14_ = 0.208, p = 0.815, left S2: F_2,14_ = 0.964, p = 0.405, right S2: F_2,14_ = 0.928, p = 0.419, S1/M1: F_2,14_ = 0.672, p = 0.527, CMA: F_2,14_ = 1.906, p = 0.185, SMAproper: F_2,14_ = 0.282, p = 0.758) (Fig. 3, bottom row).

## Discussion

The present study investigated the potential involvement of supraspinal structures in the control of muscle activation required for weight-bearing during walking. Task-related BOLD signal changes associated with *active* and *passive* stepping inside the stepping robot MARCOS were studied at three significantly different levels of load against the feet simulating vertical ground reaction forces similar to those during ground-level gait. We demonstrated overlapping activation in S1/M1 across all load levels in both conditions. The whole brain group analyses did not reveal statistically significant differences of activations in sensorimotor areas of the brain between load levels in the *active* or *passive* condition. This finding was confirmed by the subsequent ROI analysis.

### Performance of the Robot and the Participants

The analysis of motor performance metrics did not reveal a significant effect of *load level* for the performance metrics *knee amplitude* and *stepping frequency.* Motor performance was hence well matched in terms of movement extent and rhythm in both conditions by means of the stepping robot MARCOS. This is also supported by the congruence of knee position profiles across load levels (Fig. 1). At the same time, the robot successfully rendered significantly different loads against the foot soles of the participants, as a significant effect of *load level* was detected during *active* and *passive* stepping. In general, the variability of the delivered loads was higher during *active* than during *passive* stepping (shaded areas in Fig. 1, bottom row). This might be explained by the fact that during *active* and *passive* movements the robot was governed by two distinct controllers with different accuracy in force control (Hollnagel et al. 2011, 2013).

Despite the use of a robotic device, the measured mean peak interaction forces, as rendered to the participants, deviated from the values specified for each participant individually at the beginning of the experiment. At *load level 0* the robot was programmed to render 0 %-BW of additional load to the feet, however, despite the zero-force control, the measured mean peak interaction forces reached almost 10 %-BW. These undesired forces are created by intrinsic friction of the system, and cannot be eliminated because the pneumatic cylinders at the feet can only push against the foot sole, but not pull due to safety reasons.

### Similarities and Differences in Brain Activation across Load Levels

The set of supraspinal areas activated by *active* and *passive* stepping across load levels in the present study is largely in agreement with previous reports of multi-joint lower limb motor control during gait-like movements. During active and passive pedaling and stepping movements in the supine position, activation of bilateral S1/M1, SMA-proper and the cerebellar vermis has been previously reported using positron emission tomography (Christensen et al. 2000) and fMRI (Jaeger et al. 2014; Mehta et al. 2009, 2012).

During *active* movements at *load level 40,* several clusters of significant activation were observed deep within the Sylvian fissure, which were not significant during the other two load levels. Firstly, two bilateral clusters were located in the posterior fronto-parietal operculum with local peak activations centered in area OP1 in the left, and OP2 (extending into OP1) in the right hemisphere. According to (Eickhoff et al. 2007), these activations correspond to the functional area S2. Intriguingly, activation of S2 was not reported in the pedaling studies of (Christensen et al. 2000; Mehta et al. 2009, 2012). Secondly, significant activation of the bilateral dorsal-posterior anterior insula has been found for *active* stepping only at the highest load level. The peak coordinates of these clusters are compatible with the results of a recent meta-analysis of the topographical organization of the anterior insular cortex during hand and leg motor tasks (Mutschler et al. 2009). The reported foci, slightly anterior to the sulcus centralis insulae, are also found in the present study as bilateral insular activity. Activity in the anterior insula was not found during *passive* stepping at *load level 40*. These differences of activations between load levels suggested by the qualitative comparison of the activation maps indicate a modulation of brain activation by the load. However, the relevance of these between-load level differences in the sensorimotor system should be interpreted with caution considering that they did not survive statistical testing by the rmANOVAs, and the relatively small number of participants.

### Modulation of the BOLD-Signal by the Load Level

In contrast to our initial hypothesis, the present study did not reveal any significant effect of *load level* on  %-signal change in any of the ROIs during both stepping conditions, despite the provision of significant load input to the lower limbs. This is puzzling considering previous upper limp studies showing that activation in the S1/M1-area is highly correlated with electromyographic (EMG) activation and force output of hand and upper arm muscles (Keisker et al. 2010; Siemionow et al. 2000). Yet, our finding is in agreement with the pedaling study of (Christensen et al. 2000) reporting three different potential explanations for the lack of significant differences between loads observed in their study: (1) the range of investigated loads was not large enough for effects to occur; (2) the chosen methodology lacked the necessary sensitivity for effects to be revealed; or (3) the control of load-related aspects of walking occurs without involvement of the supraspinal centers. As these three rationales might also account for the lack of effects in the current study, they are further discussed in the following:

1. Insufficient increase of load

In the study of (Christensen et al. 2000), the load was increased by approximately 8 %-BW (assuming an average BW of 70 kg), however a correlating increase in regional CBF was not found. Eight percent BW is in the realm of the inter-step variability of vertical ground reaction force during level walking (Winter 1984), hence the effects of load in the study of (Christensen et al. 2000) might have been masked by the noise inherent to human lower limb motor control. In a study by Ivanenko et al., walking with only 5 % of BW already provided sufficient sensory afferences to elicit EMG-activity patterns in anti-gravity muscles of the legs, which were similar to those during walking without any BWS, if at a smaller amplitude (Ivanenko et al. 2002). Hence, already small changes of peripheral stimulation may elicit muscle activity during walking. In the fNIRS study of (Miyai et al. 2006) a load difference of 10 %-BW during walking led to a change in the level of brain activation in healthy participants and stroke patients. In the present study, the mean level-to-level increase ranged between 12 % (*active*) and 15 %-BW (*passive*), which is 50–100 % above the natural step-to-step variability of ground-level gait (Winter 1984). It is therefore reasonable to conclude that the force increments applied in the present report were sufficient to elicit differential afferent feedback from load sensitive receptors across load levels.

2) Insufficient sensitivity of the applied methodology

It cannot be entirely ruled out, that true effects of load in the present study were masked by the insufficient sensitivity of the applied imaging methodology. Several factors might have limited the sensitivity of the present investigation: First, the size of the final study sample (n = 8) was small, as a considerable amount of data (8 out of 16 participants) had to be excluded from the analysis due to excessive task-induced head motion, which occurred especially at the higher load levels. Head motion is a known issue of fMRI experiments involving movements of the lower limbs and cannot be completely eliminated by the sparse sampling imaging protocol. In an attempt to increase the sample size, we also carried out the ROI-analysis for five additional participants with head motion below one voxel size instead of the more stringent threshold of half voxel size. The addition of these participants to the study sample introduced additional variance to the data, the mean values were however not affected. We therefore decided to report the results using the more rigorous threshold despite the reduction of the study sample to eight participants. To prevent such extensive loss of data in future fMRI investigations using MARCOS, we suggest to apply prospective motion correction during functional image acquisition (Ooi et al. 2011).

Second, the applied sparse sampling image acquisition acquired the BOLD-signal only after cessation of the task. Some of the evoked hemodynamic response might not have been fully captured by the delayed acquisition of the functional images. However, sparse sampling image acquisition has been shown to be equally effective as continuous image acquisition (Nebel et al. 2005). The sensitivity of the sparse sampling approach is suggested to be further increased by consideration of individual peak latencies of the hemodynamic response during data analysis, or also by increasing the number of averaged trials (Nebel et al. 2005). An increase of the number of trials would also increase the length of the experiment and might not be optimal when investigating patients, particularly under the restrictive conditions of the robot.

Third, the ROIs for the extraction of  %-signal change in the current experiment should probably include the ‘leg-area’ of the sensorimotor areas, i.e., the areas activated by whole-leg movements, since stepping inside MARCOS can be seen as a combination of movements about the hip, knee, and ankle joints. To our knowledge, there is currently no report regarding the stereotactic coordinates of a ‘leg-area’ in any region of the brain. Therefore, spherical ROIs were built comprising of the stereotactic coordinates of isolated unilateral ankle and knee movements, as reported by (Kapreli et al. 2006), and then combined into one bilateral ROI per anatomical region, resulting in four spheres per ROI. The movement about the hip joint might hence be somewhat under-represented in the chosen ROI, which in turn may have diminished the sensitivity of the presented ROI analysis.

3) No supraspinal involvement

Recent EEG literature on brain activation during treadmill walking and upright stepping revealed dynamic modulations of cortical activity over the course of each step cycle (Gwin et al. 2011; Petersen et al. 2012; Wagner et al. 2012; Wieser et al. 2010). These modulations roughly arise at the time when loading of the limb during heel-strike and unloading at toe-off occurs. A study by (Petersen et al. 2012) reported, that the electrocortical signals recorded by Cz-electrode (i.e., approximately above the motor area of the legs in the primary sensorimotor cortex) and EMG activity from M. tibialis anterior (TA) showed significant phase coherence between signals in the beta (24–40 Hz) frequency band at the end of the swing phase. A decrease of spectral power of the same frequency band under the Cz- and Pz-electrodes, followed by a subsequent increase in the same band during the stance phase of the leg has been reported by (Wagner et al. 2012). These findings suggest that oscillatory activity in the primary motor cortex drives the activation of lower limb muscles through direct corticospinal pathways in a phase-specific manner during gait. Also Gwin et al. reported modulation of the EEG frequency spectrum along the midline electrodes, however, modulations occurred at 3–24 Hz (delta-band) and 40–76 Hz (gamma) (Gwin et al. 2011). As previously reported for the upper limbs (Omlor et al. 2007), Gwin et al. interpreted the observed modulations in the gamma-band as a shift towards the rapid integration of sensory information required for the generation of appropriate motor commands during dynamic force production, as it is required for weight-bearing during the stance-phase. (Wieser et al. 2010) also reported strong cortical activity at central midline electrode Cz in the phase of the stepping cycle when the legs are reversed from flexion to extension or vice versa. However, these authors concluded that cortical input is needed for the process of reversing the direction between the flexor and extensor movement and not in the context of weight-bearing of the legs.

In summary, these recent EEG studies very strongly suggest a temporally dynamic involvement of supraspinal centers in the regulation of walking and stepping. Unfortunately, there are currently no studies available assessing the direct relation between these temporally dynamic EEG signals and walking at different levels of BWS. Task-related fMRI as used in the present study is not suitable to reveal the temporal aspects of brain activation during task execution to the same extent as EEG. The temporal resolution of fMRI is limited, firstly by the sluggish nature of the BOLD signal, secondly because signals are acquired at a low sampling rate (i.e., 3.025 s in the present study) and thirdly, because signals are averaged over the entire trial duration (i.e., 10 s in the present study). However, if the amplitude of  %-signal change was in fact modulated by the load level, differences should still be detectable when comparing means. From this perspective, and considering the results of recent work using EEG, we would hypothesize that the activity in supraspinal centers of motor control of the lower limbs is rather associated with the monitoring of basic motor programs, i.e., related to the timing of reciprocal and rhythmic activation of the muscles of both legs. The activation of muscles and its strength required for weight-bearing during stance would then be regulated by sensorimotor control centers located outside the brain and further down-stream, e.g., in the brainstem (Jahn et al. 2008) or in the spinal cord (Dietz 1998; Duysens et al. 2000). An involvement of these structures could explain the absence of statistically significant differences in the degree of supraspinal activations between loads levels in the present study. Since the brainstem structures were not completely covered in all participants by the applied whole-brain fMRI sequence, brainstem structures were not analyzed in the context of this study. Furthermore, BOLD imaging of the brainstem is challenging due to its small size and proximity to structures of high magnetic susceptibility (Harvey et al. 2008).

In conclusion, our results show that the MR-compatible stepper MARCOS enables the delivery of external loads at different levels during task-related fMRI-experiments. However, the investigation of brain activation related to weight-bearing of the lower limbs remains challenging, as task-induced head motion continues to be an unresolved issue with conventional imaging techniques. In consequence, only data from a small number of participants could be analyzed in the present study. Nevertheless, the presented results add compelling evidence to the notion that loading of the lower limbs during stepping does not modulate the level of brain activation (i.e.,  %-signal change) in the investigated cortical and sub-cortical sensorimotor areas. The current findings should be transferred to clinical populations with much caution. The execution of stepping movements is highly automatized in healthy individuals, whereas in neurologic patients with supraspinal pathology the same type of movement may lead to differential supraspinal involvement as dysfunctions may occur at many levels of the lower limb motor control hierarchy. From this perspective, the present study demonstrates merely the feasibility of investigations of the effects of load bearing on brain activation, and it may serve as guide for future investigations on changes of supraspinal activation in specific patient populations undergoing gait rehabilitation at different levels of BWS.
